# Expression profiling suggests the involvement of hormone-related, metabolic, and Wnt signaling pathways in pterygium progression

**DOI:** 10.3389/fendo.2022.943275

**Published:** 2022-09-14

**Authors:** Jiarui Li, Tianchang Tao, Yingying Yu, Ningda Xu, Wei Du, Mingwei Zhao, Zhengxuan Jiang, Lvzhen Huang

**Affiliations:** ^1^ Department of Ophthalmology, Peking University People’s Hospital Eye diseases, and Optometry Institute, Beijing, China; ^2^ Beijing Key Laboratory of Diagnosis and Therapy of Retinal and Choroid Diseases, Peking University People’s Hospital, Beijing, China; ^3^ College of Optometry, Peking University Health Science Center, Beijing, China; ^4^ Department of Ophthalmology, The Second Affiliated Hospital of Anhui Medical University, Hefei, China

**Keywords:** Pterygium, microarray, ocular surface disease, conjunctiva, hormone, metabolism

## Abstract

**Background:**

Pterygium is an ocular surface disease that can cause visual impairment if it progressively invades the cornea. Although many pieces of research showed ultraviolet radiation is a trigger of pterygium pathological progress, the underlying mechanism in pterygium remains indistinct.

**Methods:**

In this study, we used microarray to evaluate the changes of transcripts between primary pterygium and adjacent normal conjunctiva samples in China. Then, we performed Gene Ontology (GO) and Kyoto Encyclopedia of Genes and Genomes (KEGG) functional enrichment analyses. Moreover, we constructed protein-protein interaction (PPI) and miRNA-mRNA regulatory networks to predict possible regulatory relationships. We next performed gene set enrichment analysis (GSEA) to explore the similarities and differences of transcripts between Asian studies from the Gene Expression Omnibus database. Furthermore, we took the intersection of differentially expressed genes (DEGs) with other data and identified hub genes of the development of pterygium. Finally, we utilized real-time quantitative PCR to verify the expression levels of candidate genes.

**Results:**

A total of 49 DEGs were identified. The enrichment analyses of DEGs showed that pathways such as the Wnt-signaling pathway and metabolism-related pathways were upregulated, while pathways such as hormone-related and transcription factor-associated pathways were downregulated. The PPI and miRNA-mRNA regulatory networks provide ideas for future research directions. The GSEA of selecting Asian data revealed that epithelial-mesenchymal transition and myogenesis existed in the pathology of pterygium in the Asian group. Furthermore, five gene sets (interferon-gamma response, Wnt beta-catenin signaling, oxidative phosphorylation, DNA repair, and MYC targets v2) were found only in our Chinese datasets. After taking an intersection between selecting datasets, we identified two upregulated (*SPP1* and *MYH11*) and five downregulated (*ATF3*, *FOS*, *EGR1*, *FOSB*, and *NR4A2*) hub genes. We finally chose night genes to verify their expression levels, including the other two genes (*SFRP2* and *SFRP4*) involved in Wnt signaling; Their expression levels were significantly different between pterygium and conjunctiva.

**Conclusions:**

We consider hormone-related, metabolic, and Wnt signaling pathways may be important in the pathology of pterygium development. Nine candidate genes we identified deserve further study and can be potential therapeutic targets.

## 1 Introduction

Pterygium is a benign ocular surface disease that typically appears as a fibrovascular conjunctiva thickening from the nasal side and may progressively extend onto the cornea, causing visual impairment (1, 2). The incidence of pterygium was 1.4% in the Asian population and 2.1% in the Chinese (3). Ultraviolet (UV) radiation, male gender, older age, ethnicity, outdoor occupation, and air pollution were associated with the occurrence and development of pterygium (3–5). Pterygium excision with conjunctival autograft and adjuvant therapy is the conventional treatment (6). Using fibrin glue in place of sutures for attaching conjunctival autograft during the surgery could further reduce the recurrence rate (0-4.5%) and surgical duration and help recover the cornea (7–9). Although many efforts have made progress, more surgical and non-surgical approaches are needed to be explored to decrease the recurrence further.

Currently, the underlying mechanism in pterygium remains indistinct. UV radiation is a well-recognized major cause of pterygium, and many research studies believe it triggers oxidative stress leading to DNA damage and subsequently stimulates multiple pathogenic factors (such as tumor suppressor P53, growth factors, pro-inflammatory cytokines, and extracellular matrix modulation) to induce a hyperproliferative state (10–15). On the other hand, hereditary factors and viruses (e.g., human papillomavirus) are also reported to contribute to the disease, so pterygium is considered an outcome of a combination of genetic and environmental factors (1, 15). Moreover, pterygium is regarded as a limbal stem cell disorder with precancerous features (16). In short, despite many studies on pterygium, there are lots of unsolved puzzles that need to be unraveled.

Many studies have utilized transcriptome technologies to identify changes in transcripts of pterygium in recent years (17–23). They have greatly advanced the research on pterygium and identified several groups of abnormally expressed genes in pterygium, such as keratins (*KRT3*, *KRT4*, *KRT6B*, *KRT13*, *KRT14*, *KRT16*, and *KRT24*); S100 calcium-binding proteins (*S100A8*, *S100*A9, *S100A11*, and *S100P*); collagens (*COL1A1*, *COL8A1*, and *COL10A1*). Furthermore, other studies have reported the involvement of various matrix metalloproteinases in the progression of pterygium (*MMP1*, *MMP2*, *MMP3*, *MMP9*, and *MMP13*) *(* 24–26). On the other hand, it has been reported that the Chinese have a higher incidence of the disease than the whole Asian population (3), so the transcriptomic information needs to be more abundant. Moreover, identifying the similarities and differences of transcripts between Asian studies is necessary to reveal the underlying mechanisms of pterygium.

In this study, we used microarray to evaluate the changes of transcripts between primary pterygium samples and adjacent normal conjunctiva samples in China and perform multiple bioinformatic analyses. Next, we took the intersection of differentially expressed genes with other data in Asian countries and identified hub genes. Finally, we verified the expression levels of candidate genes from microarray data. Our study hopes to provide more evidence of the progression of pterygium and find potential treatment targets.

## 2 Material and methods

### 2.1 Specimens preparation

This study approval was provided by the Ethics Committee of the second affiliated hospital of Anhui Medical University in accordance with the Helsinki Declaration of 1975. Sixteen nasal primary pterygium samples and fourteen adjacent normal conjunctiva samples were obtained from 16 participants (aged 47-70 years, five males and 11 females) through pterygium excision surgery. All the procedures were performed by an experienced ophthalmologist in the second affiliated hospital, Anhui Medical University. All of the participants in this study signed the informed consent before they were enrolled in the study. After pterygium excision, the whole part of the tissues was immediately placed in RNALater™ RNA Stabilization Reagent for Animal Tissue (Beyotime, China, R0118) and infiltrated overnight at four degrees, then all of the tissues were stored at a −80 °C refrigerator until RNA extraction.

### 2.2 RNA isolation

The total RNA of the tissues was isolated using TRIzol™ Reagent (Invitrogen, USA, 15596026) according to the manufacturer’s protocol. For microarray analysis, two pairs of pterygium and adjacent normal conjunctiva samples were used. The other tissues were used in real-time quantitative polymerase chain reaction (qPCR). The quality control and quantification of RNA were performed by electrophoresis on 1% agarose gels and NanoDrop 2000 (Thermo Fisher Scientific, USA).

### 2.3 Microarray analysis

After the quality test of RNA, gene expression profiling was conducted by Shanghai Baygene Biotechnology Co.Ltd (Shanghai, China) using GeneChip^®^ Human Transcriptome Array 2.0 (HTA 2.0). cDNA was synthesized, amplified, fragmented, and labeled for hybridization using the GeneChip^®^ WT PLUS Reagent Kit (Affymetrix, USA, 902280) from 2 ugs isolated RNA following the manufacturer’s protocols. Then, GeneChip^®^ Expression Wash, Stain, and Scan were performed following protocol from GeneChip^®^ Hybridization Wash and Stain Kit (Affymetrix, USA, 900720). Probe cell intensity data (CEL) from microarrays are analyzed and normalized by Signal Space Transformation Robust Multichip Analysis (SST-RMA) algorithm in the Affymetrix^®^ Expression Console™ software (v1.4). The normalized data were then subjected to R package limma (v3.42.2) to identify differentially expressed genes (DEGs) (27). Genes with |log2FC| >1 and p-value < 0.05 were identified as DEGs. Volcano plot visualization was exerted by ggplot2 (v3.3.0). Next, the DEGs were submitted to execute the Gene Ontology(GO) and the Kyoto Encyclopedia of Genes and Genomes(KEGG) functional enrichment analyses utilizing the R package ClusterProfiler (v3.14.3) (28). For Protein-protein interaction (PPI) network construction, we used the STRING database (https://string-db.org/cgi/input.pl) to recognize the possible connections between DEGs, the data exported from the STRING database was visualized by Cytoscape (v3.8.2), and the MCODE (v2.0.0) plugin of Cytoscape was applied to identify a key module of PPI network (29).

### 2.4 Data collection

For further bioinformatic analyses, we collected miRNA and microarray data from PubMed and the Gene Expression Omnibus database (GEO, www.ncbi.nlm.nih.gov/geo) based on our selection criteria. As for miRNA selection, we used “Pterygium” and “miRNA” as keywords to search on Pubmed and screen research from Asia. Next, we included 14 miRNAs that were validated by qPCR or could be found in at least two microarray data. The information on miRNA enrolled in our study is listed in **Table 1** (30–37). The selection of microarray datasets for this study was based on screening the GEO database, and datasets from Asia were selected. The details of these microarray datasets are shown in **Table 2**.

**Table 1:** The information on miRNA for further analysis.

miRNA	Change	p.value	Country	Verification	Source
hsa-miR-221	Up	<0.0001	China	qPCR	PMID:25053875 (30)
hsa-miR-21	Up	<0.01	China	qPCR	PMID:30967746 (31)
hsa-miR-143-3p	Up	<0.005	China	qPCR	PMID:29360447 (32)
hsa-miR-145-5p	Up	<0.05	China	qPCR	
hsa-miR-30a-5p	Up	0.043	China	qPCR	PMID:32867783 (33)
hsa-miR-143-5p	Up	0.001		qPCR	
hsa-miR-199-3p	Up	0.001		qPCR	
hsa-miR-199-5p	Up	0.002		qPCR	
hsa-miR-486-3p	Up	0.001		qPCR	
hsa-miR-215	Down	0.028	Singapore	qPCR
hsa-miR-200a	Down	0.015	China	qPCR	PMID:26995143 (35)
hsa-miR-218-5p	Down	<0.01	China	qPCR	PMID:30243568 (36)
hsa-miR-122	Down	<0.05	China	qPCR	PMID:27415790 (37)
hsa-miR-1298-5p	Up	0.046	Singapore/China	–	From the intersection of GSE21346 and PMID:27415790

**Table 2:** The descriptions of GEO datasets used to perform subsequent analyses.

Datasets ID	Country	Samples	Platforms	Microarray types	DEGs	
GSE83627	Singapore	4 Conjunctiva cases 4 Pterygium cases	GPL14550 SurePrint G3 Human GE 8x60K Microarray	mRNA	Up	523
					Down	0
GSE51995	Singapore	4 Conjunctiva cases 4 Pterygium cases	GPL14550 SurePrint G3 Human GE 8x60K Microarray	mRNA	Up	508
					Down	231
GSE2513	Singapore	4 Conjunctiva cases 8 Pterygium cases	GPL96 Affymetrix Human Genome U133A Array	mRNA	Up	114
					Down	73
GSE151872	Japan	1 Conjunctiva cases 3 Pterygium cases	GPL17077 Agilent-039494 SurePrint G3 Human GE v2 8x60K Microarray	mRNA	Up	–
					Down	–
GSE21346	Singapore	3 Conjunctiva cases 3 Pterygium cases	GPL7723 miRCURY LNA microRNA Array, v.11.0	microRNA	Up	4
					Down	1

GEO, Gene Expression Omnibus; DEGs, Differentially expressed genes.

### 2.5 The miRNA-mRNA regulatory network construction

All enrolled miRNAs were submitted to ENCORI (the encyclopedia of RNA Interactomes, https://starbase.sysu.edu.cn/) to predict and acquire their targeted mRNAs (38). The ENCORI could also connect to seven other target mRNA-predicted programs (microT, miRanda, miRmap, PITA, RNA22, PicTar, and TargetScan); the mRNAs we selected were overlapped in at least four predicted programs. The miRNAs usually perform as negative regulators to affect the expression of the target mRNAs, upregulated miRNA led to downregulated target mRNA and vice versa. Considering such a regulatory pattern, we took the intersection of the acquired mRNAs with our microarray data based on the expression change direction. The visualization of the miRNA-mRNA regulatory network was carried out by TBtools (v1.0986988) (39).

### 2.6 Gene set enrichment analysis

The gene set enrichment analysis (GSEA) based on Hallmark gene sets in the molecular signatures database (v7.5.1) was performed by GSEA software (v4.2.3). The results of GSEA were subjected to TBtools to visualize as a heat map, and data was clustered using Euclidean distances with complete hierarchical clustering.

### 2.7 Hub genes identification and enrichment analysis

To identify hub genes, we processed microarray datasets GSE83627, GSE51995, and GSE2513 by limma (v3.42.2). Besides, the microarray dataset GSE151872 only has one pterygium sample and is not applicable for limma’s protocol, so we discarded this dataset to recognize hub genes. We took the intersection of DEGs from our microarray data and the above datasets, then submitted the results to the STRING database to perform further enrichment analysis. The visualizations were conducted by TBtools, Cytoscape, and ggplot2.

### 2.8 Real-time quantitative polymerase chain reaction

cDNA was synthesized from a total of 1 µg RNA with the ReverTra Ace^®^ qPCR RT Master Mix with gDNA Remover (Toyobo Co., Ltd., Japan, Code No.FSQ-301). Real-time quantitative polymerase chain reactions (qPCR) were carried out using SYBR^®^ Green Real-time PCR Master Mix (Toyobo Co., Ltd., Japan, Code No. QPK-201) with Roche LightCycler 480 (Roche Diagnostics Lid. Switzerland). Evaluating relative mRNA expression by using *GAPDH* as endogenous control *via* the 2^−△CT^ methods. The experiments were repeated using three technical replicates. **Table 3** lists all primers.

**Table 3:** Primers for real-time quantitative PCR.

Gene Symbol	EnsemblID	Sequence (5’ -> 3’)		Amplicon Size
*SPP1*	ENSG00000118785	Fp	TTCTGATTGGGACAGCCGTG	199bp
		Rp	TCTCATCATTGGCTTTCCGCT	
*MYH11*	ENSG00000133392	Fp	CGCCAAGAGACTCGTCTGG	129bp
		Rp	TCTTTCCCAACCGTGACCTTC	
*ATF3*	ENSG00000162772	Fp	CCTCTGCGCTGGAATCAGTC	111bp
		Rp	TTCTTTCTCGTCGCCTCTTTTT	
*FOSB*	ENSG00000125740	Fp	GCTGCAAGATCCCCTACGAAG	249bp
		Rp	ACGAAGAAGTGTACGAAGGGTT	
*FOS*	ENSG00000170345	Fp	GGGGCAAGGTGGAACAGTTAT	126bp
		Rp	CCGCTTGGAGTGTATCAGTCA	
*NR4A2*	ENSG00000153234	Fp	GCACTCCGGGTCGGTTTAC	129bp
		Rp	GCCACGTAGTTCTGGTGGAA	
*EGR1*	ENSG00000120738	Fp	GGTCAGTGGCCTAGTGAGC	149bp
		Rp	GTGCCGCTGAGTAAATGGGA	
*SFRP2*	ENSG00000145423	Fp	ATGCTTGAGTGCGACCGTTT	99bp
		Rp	TACCTTTGGAGCTTCCTCGG	
*SFRP4*	ENSG00000106483	Fp	ACGAGCTGCCTGTCTATGAC	99bp
		Rp	TGTCTGGTGTGATGTCTATCCAC	
*GAPDH*	ENSG00000111640	Fp	ACAACTTTGGTATCGTGGAAGG	101bp
		Rp	GCCATCACGCCACAGTTTC	

Fp, Forward primer; Rp, Reverse primer.

### 2.9 Statistical analysis

Analyses were performed and visualized by GraphPad Prism 8.3.0. After failing to pass the Shapiro-Wilk normality test, then comparisons of data were made using the Mann–Whitney nonparametric test. A p-value of <0.05 was considered a significant difference.

## 3 Results

### 3.1 Results of microarray data analysis

#### 3.1.1 The differential gene expression analysis

**Figure 1:**
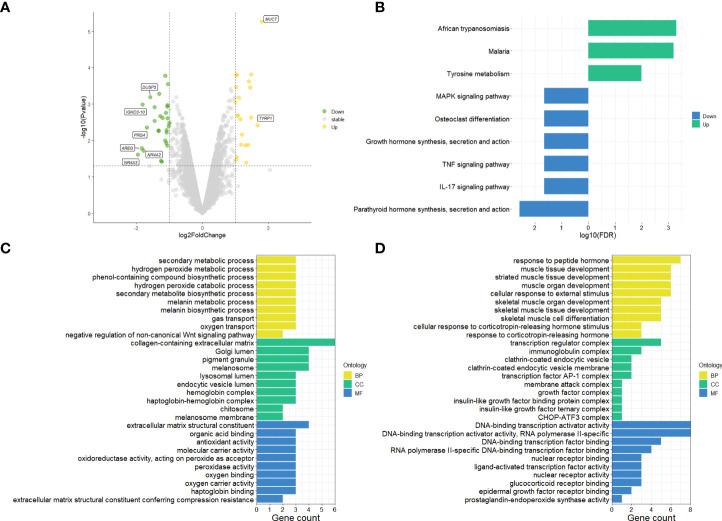
Results of microarray data analysis. **(A)** Volcano plot of microarray data, upregulated genes fill with yellow while downregulated genes fill with green. Grey dots mean genes are stable. The dotted horizontal lines indicate a p.value of 0.05, and the vertical lines indicate a log2FC of 1. Genes (|log2FC| > 1.5) labeled with their gene symbol are shown in the Figure. **(B)** KEGG analysis of DEGs of pterygium. The green color indicates upregulation, and blue indicates downregulation. **(C)** Top 30 upregulated GO terms for DEGs in different classifications. **(D)** Top 30 downregulated GO terms for DEGs in different classifications. The selection criteria of significant pathways or GO terms was FDR < 0.05. log2FC, log2foldchange; GO, Gene Ontology; KEGG, Kyoto Encyclopedia of Genes and Genomes (http://www.genome.jp/kegg/); FDR, false discovery rate. BP, biological process; CC, cellular component; MF, molecular function.

The differential gene expression analysis identified a total of 49 DEGs (|log2FC| >1 and p-value < 0.05) on primary pterygium samples vs. adjacent normal conjunctiva samples. Among them, 21 were upregulated, and 28 were downregulated; all the DEGs and details are listed in **Supplementary Table 1**. The volcano plot (shown in **Figure 1A**) of the microarray data indicated the distribution of the DEGs. The upregulated DEGs were shown in yellow and downregulated were shown in green.

#### 3.1.2 KEGG and GO pathway enrichment analysis

To get more bioinformatic signatures of the pterygium, we next performed enrichment analyses of the DEGs. **Figure 1B** presented the results of the KEGG pathway enrichment analysis. The upregulated pathways were related to infection and metabolism, and the downregulated were associated with inflammation, hormone, and cell differentiation. As for GO analysis, a total number of 195 GO terms of upregulated DEGs and 237 GO terms of downregulated DEGs were enriched (**Supplementary Table 2** listed all the GO terms), the top 30 of them in different classifications (cellular component, molecular function, and biological process) were shown in **Figures 1C, D** for the up- and downregulated GO terms respectively. Negative regulation of the Wnt-signaling pathway, peroxide and oxygen related, and metabolism-related pathways could be found in upregulated GO terms (**Figure 1C**). Additionally, hormone and skeletal muscle related and transcription factor associated pathways were enriched in downregulated GO terms (**Figure 1D**).

#### 3.1.3 PPI network construction

**Figure 2:**
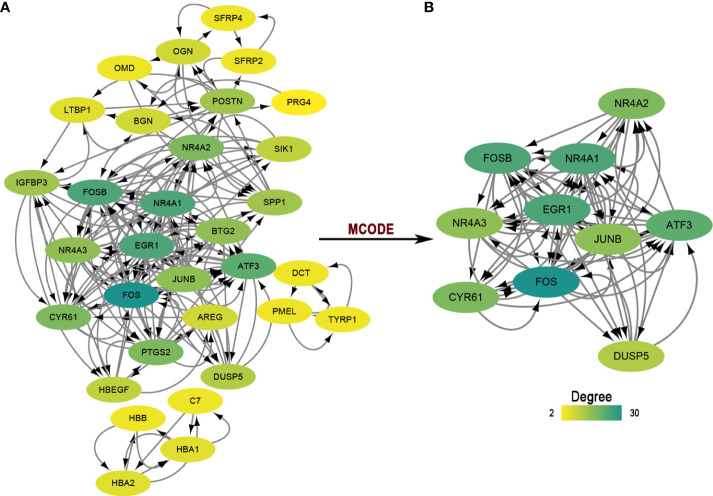
The PPI network and module analysis. **(A)** The entire PPI network of DEGs. Total of 32 nodes and 188 interacted edges in the PPI network. **(B)** A significant module of the PPI network. PPI, protein-protein interaction.

For protein-coding genes of the DEGs, we next put them into the STRING for a PPI network (**Figure 2A**). There were 32 nodes and 188 interacted edges in the PPI network. Then, we used MCODE to identify a key module for the whole network (**Figure 2B**); this cluster scoring 8.444 had ten nodes and 76 edges and might play a role in the pterygium.

### 3.2 The miRNA-mRNA regulatory network construction

**Figure 3:**
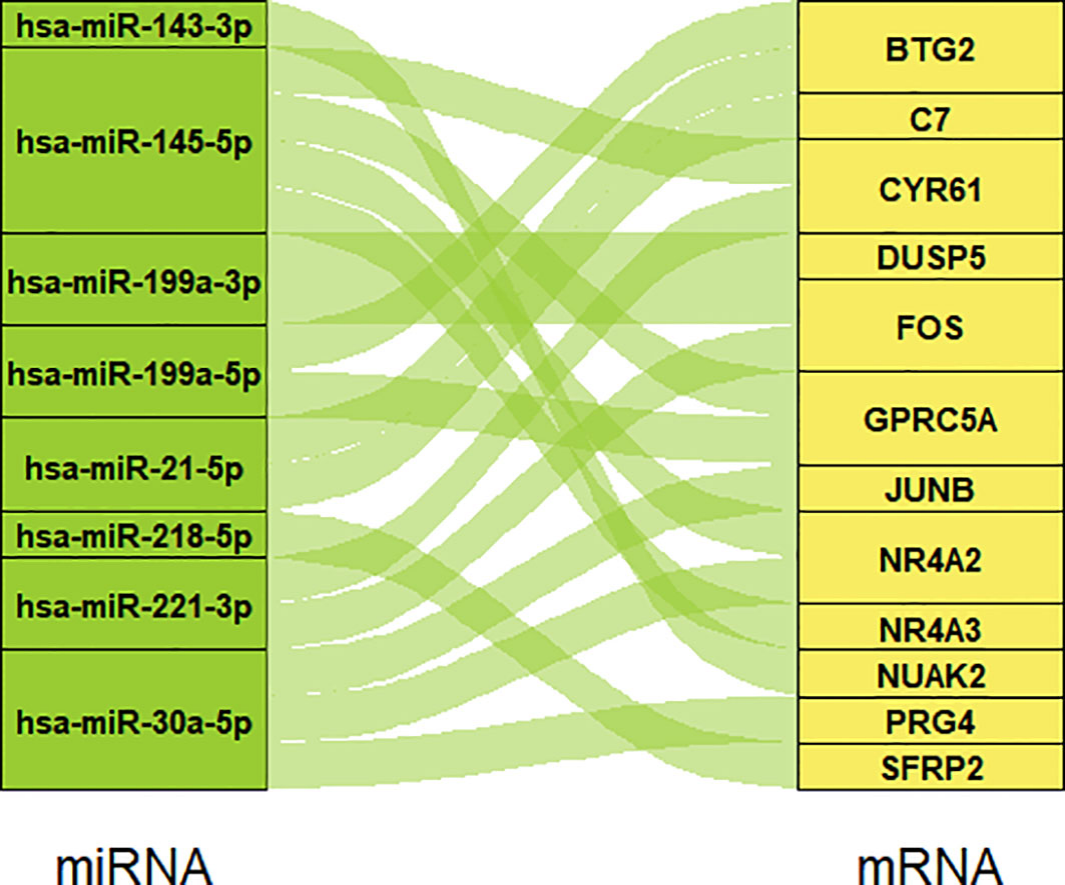
The miRNA-mRNA regulatory network. Eight miRNAs and 12 mRNAs construct 17 regulatory pairs.

We searched on PubMed using the keywords “Pterygium” AND “miRNA” for all years, then selected the miRNAs in pterygium from Asian research based on the selection criteria. **Table 1** summarizes the information on the selected miRNAs. Next, we put these miRNAs into ENCORI to predict the targeted mRNA and merged the results with our microarray data according to the screening criteria. Finally, a total of eight miRNAs and twelve mRNAs constructed 17 regulatory pairs. The miRNA-mRNA regulatory network was shown in **Figure 3**, and there existed a one-to-many regulatory relationship.

### 3.3 To compare the similarities and differences between Asian studies by gene set enrichment analysis

**Figure 4:**
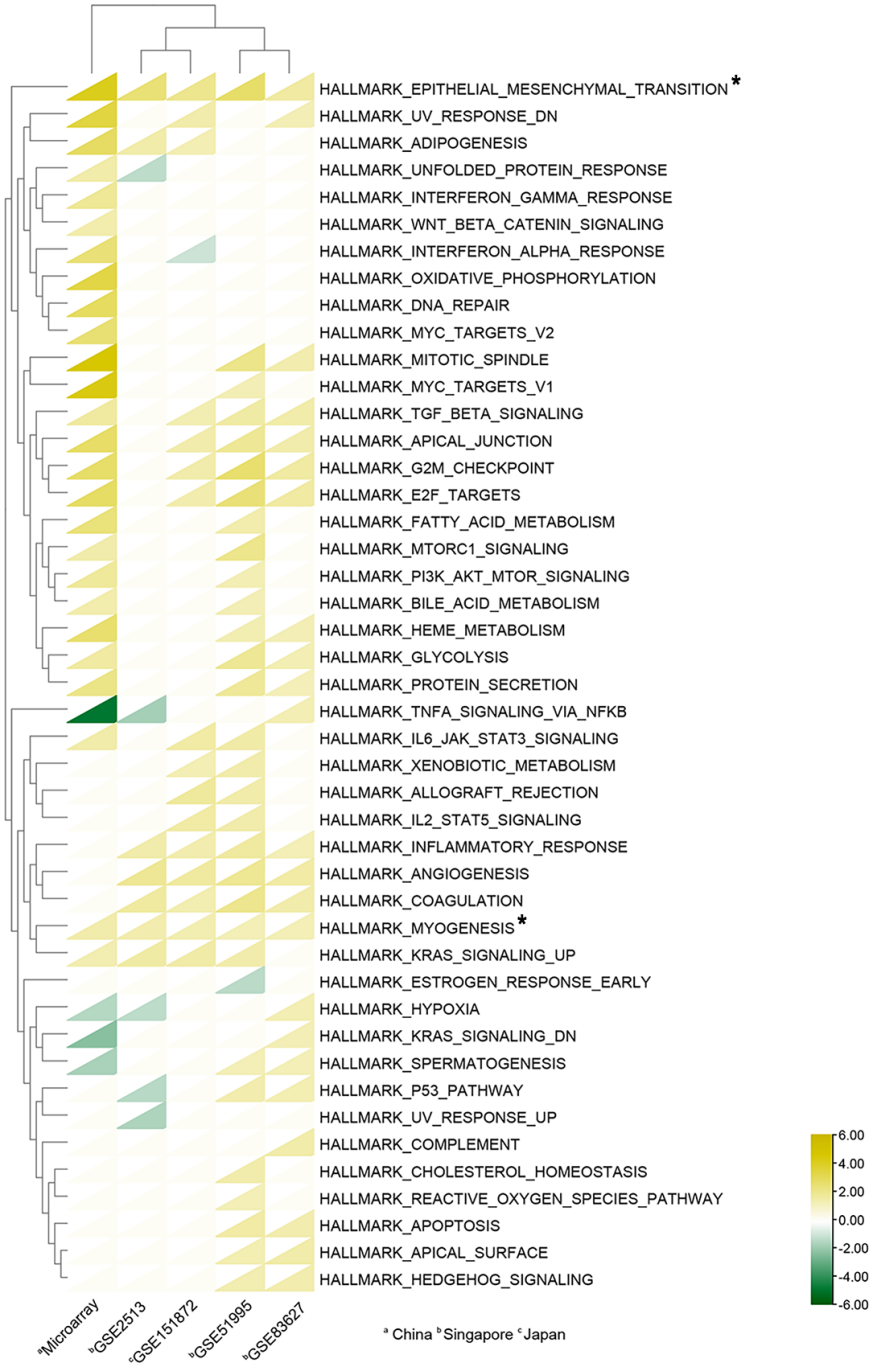
Heat map of Gene sets enriched in GSEA analysis. A total of 45 gene sets were enriched. The triangles in yellow indicate upregulated gene sets (NES > 0), while the triangles in green indicate downregulated gene sets (NES < 0); Triangles in white show no enrichment. Asterisks mean gene sets enriched in all datasets. The cluster analyses were based on Euclidean distance.

We found four datasets from Gene Expression Omnibus (GEO) in Asia (Singapore and Japan) to perform the following analysis (**Table 2**). We subjected the whole expression matrixes of our data and four datasets from GEO to GSEA software (v4.2.3), selecting Hallmark gene sets in the molecular signatures database (v7.5.1) to find similar and different biological processes of pterygium in Asia. As shown in **Figure 4**, only two gene sets were enriched in all datasets (epithelial-mesenchymal transition and myogenesis). Three gene sets were in the results of the other four datasets but not ours (inflammatory response, angiogenesis, and coagulation). Moreover, five gene sets were found only in our microarray data (interferon-gamma response, Wnt beta-catenin signaling, oxidative phosphorylation, DNA repair, and MYC targets v2). Furthermore, there were several gene sets in our data that were enriched in the other three data. Our microarray data was indicated as a separate clustering in cluster analysis based on Euclidean distance. The complete results of GSEA are demonstrated in **Supplementary Table 3**. The bigger the NES, the stronger the effect. NES > 0 meant upregulated while NES < 0 represented downregulated.

### 3.4 Identifying the hub genes by taking intersection with other microarray data

**Figure 5:**
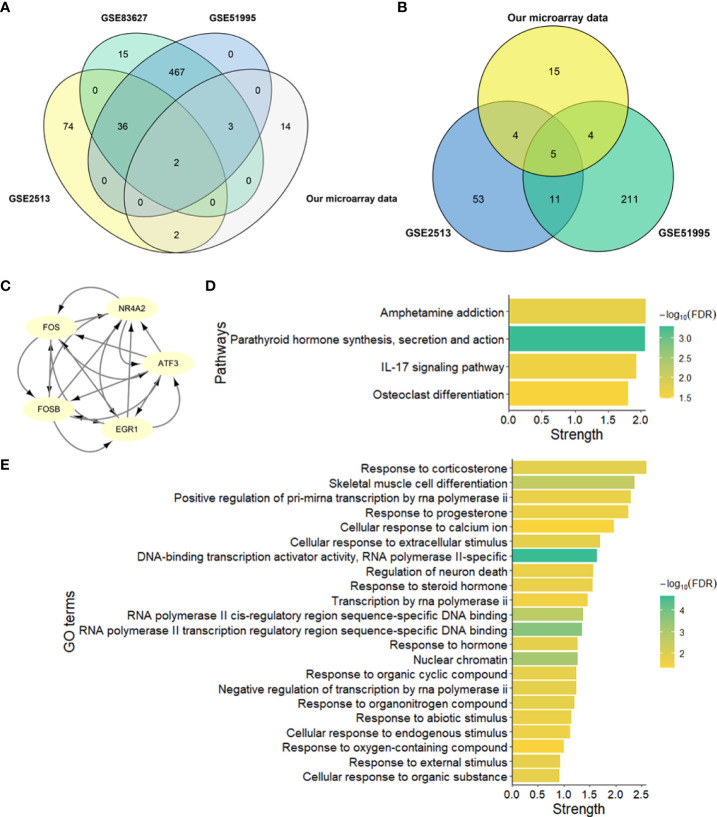
Identification and network enrichment analysis of hub genes. **(A)** Venn plot of upregulated genes of four microarray data. **(B)** Venn plot of downregulated genes of four microarray data. **(C)** PPI network of five downregulated hub genes. There are five nodes and ten edges. **(D)** KEGG pathway network enrichment analysis of five downregulated hub genes. **(E)** GO network enrichment analysis of five downregulated hub genes. Strength describes how significant the enrichment effect is.

To identify the hub genes in the development of pterygium, we took an intersection between our data and other data from GEO datasets (**Figures 5A, B**). There were only two upregulated and five downregulated genes at the intersection of four microarray datasets (**Table 4**). All of them were protein-coding genes. Strikingly, all five downregulated genes were in the key cluster that MCODE identified (**Figure 2B**), so we next put these hub genes into STRING again and performed network enrichment analysis. Two upregulated genes showed no interaction, while five downregulated genes displayed a strong connection (**Figure 5C**). Moreover, the network enrichment analysis (**Figures 5D, E**) of these five genes showed similarity with enrichment analysis of downregulated DEGs (e.g., Parathyroid hormone synthesis, secretion, and action; IL-17 signaling pathway; Osteoclast differentiation). Some pathways associated with the organic and abiotic substance or stimulus resulted from network enrichment analysis but not from the whole DEGs’.

**Table 4:** The gene cluster in the intersection of datasets.

Gene symbol	Description	Change
*SPP1*	secreted phosphoprotein 1	Up
*MYH11*	myosin heavy chain 11	Up
*ATF3*	activating transcription factor 3	Down
*FOSB*	FosB proto-oncogene, AP-1 transcription factor subunit	Down
*FOS*	Fos proto-oncogene, AP-1 transcription factor subunit	Down
*NR4A2*	nuclear receptor subfamily 4 group A member 2	Down
*EGR1*	early growth response 1	Down

### 3.5 Validation of the mRNA expression levels of candidate genes by qPCR

**Figure 6:**
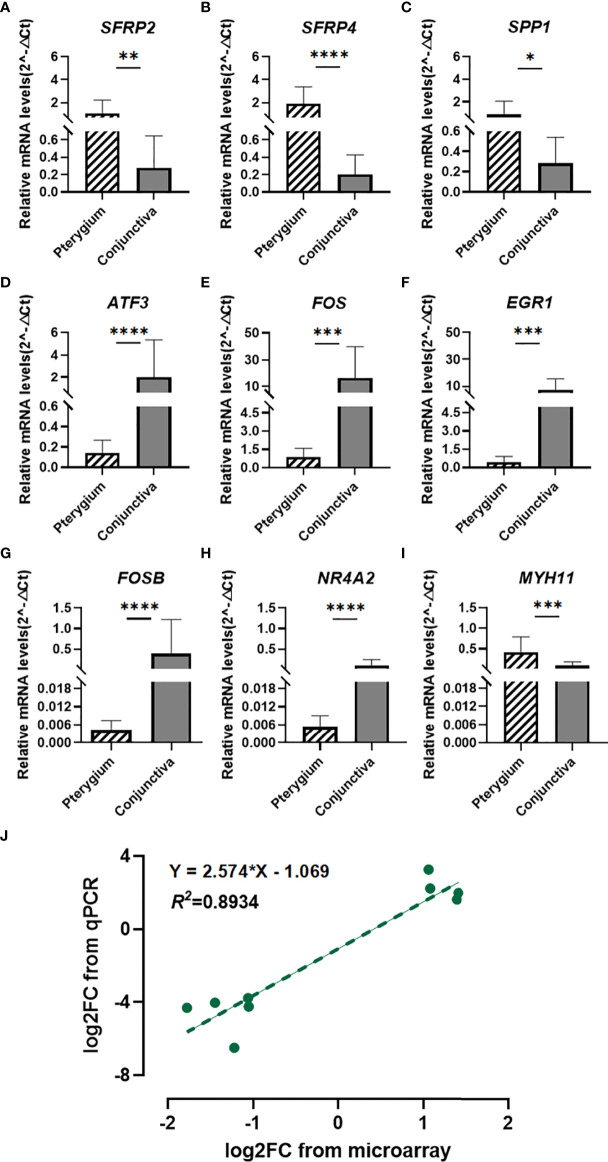
Quantitative real-time PCR verification and Correlation analysis. **(A–I)** Quantitative real-time PCR validation for *SFRP2, SFRP4, SPP1, ATF3, FOS, EGR1, FOSB, NR4A2,* and *MYH11*. n = 14 for primary pterygium samples and n = 12 for normal conjunctiva samples. *p < 0.05, **p < 0.01, ***p < 0.001, ****p < 0.0001. **(J)** Correlation analysis between microarray and qPCR data of candidate genes. Linear regression model (green dotted line): y = 2.574x - 1.069, R2 = 0.8934.

Based on the previous analysis, we chose seven hub genes to verify their expression levels. We enrolled *SFRP2* and *SFRP4* in further analysis because they were DEGs involved in Wnt signaling. As illustrated in **Figures 6A–I**, the mRNA expression levels of candidate genes (*SFRP2*, *SFRP4*, *SPP1*, *ATF3*, *FOS*, *EGR1*, *FOSB*, *NR4A2*, and *MYH11)* were significantly different in primary pterygium samples compared to conjunctiva samples. Additionally, the correlation analysis of log2 foldchange between microarray and the qPCR quantitative data demonstrated a high correlation (R^2^ = 0.8934, **Figure 6J**) which presented the accuracy of our microarray data.

## 4 Discussion

Pterygium is a common fibrovascular degeneration of conjunctiva, and ultraviolet radiation is the main cause. The best way of treating pterygium is to perform surgery with some adjuvant treatments (6, 40). Currently, the concrete mechanisms of onset and progression of pterygium still remain obscure.

To explore the underlying mechanism of pterygium, we used microarray to compare changes in transcripts between primary pterygium samples and conjunctiva samples. 49 DEGs were identified in our study, and we next conducted multiple enrichment analyses to find the potential pathways of the disease. In the results, numbers of the metabolic and biosynthetic processes were upregulated (seven of the top ten GO BP terms), and these processes represent that the cells in pterygium were actively reacting to the compounds (e.g., hydrogen peroxide) involved in them. As part of reactive oxygen species, hydrogen peroxide is responsible for oxidative stress, while oxidative stress is considered a pathogenic mechanism of pterygium (12, 41). Additionally, metabolites from healthy and diseased cells can be directly released to tear, making the difference in tear proteome between pterygium and other diseases (42). The Wnt-signaling pathway, which can control stem cell biology and growth, is also upregulated; the changes in gene expression associated with Wnt-signaling in pterygium have been reported; however, how this pathway affects the development of pterygium needs to be studied in depth (43, 44). Downregulated pathways also provide us with pieces of evidence of disease progression. Hormone-related pathways are significantly downregulated; some research reported that hormone-related growth factors and receptors are implicated in pterygium (45, 46). Even though some hormones can affect corneal morphology, physiology, and metabolism, there is few of evidence indicating that hormones directly participate in the development of pterygium (47, 48). On the other hand, long-term UVA radiation to the eyes can affect the level of corticotropin-releasing hormone in the brain (49). So, we infer that the changes in hormone-related pathways are the response to UVA radiation. Furthermore, growth factors are also downregulated. Much evidence suggests that multiple growth factors are involved in pterygium, and their expression pattern may lead to a wide variation in the growth of pterygium (15). Other downregulated pathways (e.g., tumor necrosis factor (TNF) signaling, mitogen-activated protein kinase (MAPK) signaling, and transcription factor) have strong relationships with UV radiation or pterygium (50–52). The above results, to some extent, confirmed the reliability of our microarray data.

Nowadays, the PPI network and miRNA-mRNA regulatory network have been constructed in many studies (53–55). Since our new microarray data and new miRNA data from PubMed became available, we constructed new networks based on recent data. Our PPI network and miRNA-mRNA regulatory network hope to provide a new basis for further study of pterygium. Moreover, hsa-miR-199a-3p targets *DUSP5* in our network have been confirmed in pterygium (33).

Besides performing analyses of our microarray data, we also compare our data with other Asian datasets from GEO. The hallmark gene sets include 50 gene sets, while 45 gene sets were enriched through GSEA. As displayed in **Figure 4**, epithelial-mesenchymal transition (EMT) and myogenesis are found upregulated in all datasets. EMT, playing an essential role in wound healing and tissue remodeling, is a biological process that induces the transition of polarized and immotile epithelial cells into motile mesenchymal fibroblast-like cells (56). EMT and myogenesis in our results represent epithelial cells converted to myofibroblasts, serving as a key feature of pathological tissue repair and playing an essential role in pterygium progression (33, 57). As for the differences between our data and other Asian data, we found five significant pathways only upregulated in our results: interferon-gamma response, Wnt beta-catenin signaling, oxidative phosphorylation, DNA repair, and MYC targets v2. As outlined previously, oxidative phosphorylation and DNA repair are related to oxidative stress and DNA damage which are downstream of UV radiation. Interferon-gamma response belongs to immune responses which have been associated with pterygium (58). MYC, as a proto-oncogene is also involved in the disease (59). The result of Wnt beta-catenin signaling is consistent with our previous GO analysis. On the other hand, three pathways (inflammatory response, angiogenesis, and coagulation) correlating with pterygium are not present in our data but exist in the other four datasets (15). Interestingly, three datasets show upregulation of the pathways named UV response downregulated. This result probably shows that the response to UV radiation is different between pterygium cells and healthy cells, while pterygium epithelium possibly has resistance to UV-induced apoptosis (14). In conclusion, the results of GSEA have offered a shred of potential evidence for the cause of differences in pterygium incidence in Asia. The pathways that are unique and absent in our datasets should be worth attention.

To further investigate the common mechanism of pathogenesis in pterygium, we took the intersection between our data and other GEO datasets we selected. A total of seven genes were identified as hub genes. The two upregulated genes show no interactions, and the network enrichment analysis shows no results. We later subjected them to ClusterProfiler, and the results were similar to previous GO terms that they involved (data not shown). *SPP1* encodes osteopontin playing important roles in wound healing. Osteopontin is suggested to affect metalloproteinase (MMP) secretion and influence fibroblast proliferation dependent on the growth factors (60). This process is highly concordant with mechanisms that are reported in the progression of pterygium (25). *MYH11* encoding myosin heavy chain 11 has been confirmed its involvement in several types of cancer and is probably related to metabolism-related genes (61). Five downregulated hub genes are included in the key module that MCODE identified and have interactions with each other, which means they are in the important section of disease development. These five hub genes are transcription factors. Network enrichment analysis shows they are also significantly associated with hormone-related pathways. Among them, *FOS* and *FOSB*, as members of the AP-1 transcription factor complex, were upregulated in corneal epithelial cell layers when under UV exposure for one to six hours (62). Furthermore, the c-fos mRNA level is intermediate-early induced by UV radiation in cultured pterygium epithelial cells but downregulated after 12h (52). Additionally, after applying oxidative stress to human retinal pigment epithelium, the changes of expression of *ATF3* (another member of AP-1 transcription factors), *FOS*, and *FOSB* are in a dose-dependent manner (63). Moreover, overexpression of *SPP1* in cells can downregulate the level of c-fos, and *SPP1* plays a role in wound healing (64). So, since AP-1 transcription factors are downregulated in this study, we infer that their levels depend on the balance of effects of UV exposure and wound healing. *EGR1* encodes early growth response, critically participating in neovascularization, tumor angiogenesis, and growth, together with *ATF3, which* can influence the limbal epithelial cell proliferation (65, 66). *NR4A2* is also associated with tumor proliferation, migration, and invasion (67). In summary, we speculate these hub genes are associated with pterygium proliferation and may response for resistance to UV exposure, these effects may be involved in inactivation of hormone-related pathways.

Finally, we verified the mRNA levels of nine candidate genes through qPCR. Correlation analysis shows the qPCR data are consistent with microarray data, suggesting the credibility of our results. Besides seven hub genes, we also validated two upregulated gene expressions (*SFRP2* and *SFRP4*). They are served as classical antagonists of Wnt signaling and can interact with Wnt protein directly through both autocrine and paracrine modes. In some cases, they also can promote Wnt signaling (68). Wnt signaling can control adult stem cell biology and growth (69); meanwhile, pterygium is thought to be a limbal stem cell disorder (16). The results of these two genes remind us that genes located upstream of the Wnt signaling pathway are involved in the progression of pterygium and probably can be used as therapeutic targets. Additionally, the Wnt beta-catenin signaling is the unique gene set in the results of GSEA in our data, so further in-depth studies are required, especially for the Chinese. Three hub genes (*FOS*, *NR4A2*, and *SFRP2*) are also in our miRNA-mRNA network; the predicted regulatory relationships are worthy of future research.

There were some deficiencies in our study. 1) Our sample size was insufficient and may cause a potential bias. 2) The validations of protein levels of our candidate genes were not conducted. The following study is currently ongoing to remedy the shortcomings of this study.

In conclusion, we consider hormone-related, metabolic, and Wnt signaling pathways may be important in developing pterygium besides its classical mechanisms. Nine candidate genes we identified deserve further study and can be potential therapeutic targets. In short, our findings shed important light on the further study of pterygium.

## Data availability statement

The data presented in the study are deposited in the GEO repository, accession number GSE208384.

## Ethics statement

The studies involving human participants were reviewed and approved by The Ethics Committee of The Second Affiliated Hospital of Anhui Medical University. The patients/participants provided their written informed consent to participate in this study.

## Author contributions

JL and TT wrote this manuscript and performed bioinformatic analyses. YY, NX, and WD conducted data collection and experiment process. ZJ carried out a surgical procedure. MZ and LH provided the idea for this study, and LH revised this manuscript. JL and TT have contributed equally to this work. All authors contributed to the article and approved the submitted version.

## Funding

This work was supported by the Beijing-Tianjin-Hebei Special Project (Grant Number J200014), the Science and technology innovation project of the Chinese Academy of medical sciences(2019-RC-HL-019), the National Natural Science Foundation of China (Grant Number 81670870). National Key R&D Program of China (Grant No. 2020YFC2008200). The funders had no role in the study design, data collection, analysis, decision to publish, or manuscript preparation.

## Acknowledgments

We are grateful to all the participants and their families for their generosity and support.

## Conflict of interest

The authors declare that the research was conducted in the absence of any commercial or financial relationships that could be construed as a potential conflict of interest.

## Publisher’s note

All claims expressed in this article are solely those of the authors and do not necessarily represent those of their affiliated organizations, or those of the publisher, the editors and the reviewers. Any product that may be evaluated in this article, or claim that may be made by its manufacturer, is not guaranteed or endorsed by the publisher.
